# Influence of Smartphones and Software on Acoustic Voice Measures

**DOI:** 10.5195/ijt.2016.6202

**Published:** 2016-12-15

**Authors:** ELIZABETH U. GRILLO, JENNA N. BROSIOUS, STACI L. SORRELL, SUPRAJA ANAND

**Affiliations:** 1 WEST CHESTER UNIVERSITY OF PENNSYLVANIA, DEPARTMENT OF COMMUNICATION SCIENCES AND DISORDERS, WEST CHESTER, PENNSYLVANIA, USA; 2 UNIVERSITY OF SOUTH FLORIDA, DEPARTMENT OF COMMUNICATION SCIENCES AND DISORDERS, TAMPA, FLORIDA, USA

**Keywords:** Acoustic, Applications, Apps, Smartphones, Voice

## Abstract

This study assessed the within-subject variability of voice measures captured using different recording devices (i.e., smartphones and head mounted microphone) and software programs (i.e., Analysis of Dysphonia in Speech and Voice (ADSV), Multi-dimensional Voice Program (MDVP), and Praat). Correlations between the software programs that calculated the voice measures were also analyzed. Results demonstrated no significant within-subject variability across devices and software and that some of the measures were highly correlated across software programs. The study suggests that certain smartphones may be appropriate to record daily voice measures representing the effects of vocal loading within individuals. In addition, even though different algorithms are used to compute voice measures across software programs, some of the programs and measures share a similar relationship.

Voice disorders are the most common communication disorder across the lifespan, impacting 7.5 million people in the USA (NIDCD, 2014) with one in 13 adults affected annually (Bhattacharyya, 2014). In addition, there is also evidence that voice problems may negatively influence health-related quality of life (Cohen, 2010). In standard clinical practice, patients with voice problems visit a multidisciplinary team consisting of a speech-language pathologist (SLP), otolaryngologist, and singing voice specialist for pre- and post-treatment evaluations. This approach only provides two “snapshots” of perceptual, acoustic, and aerodynamic measures. Such snapshots of vocal evaluation cannot fully capture the day-to-day effects of vocal loading (i.e., repeated vocal fold posturing or excessive tissue vibration), especially in professional voice users. Indeed, the effects of vocal loading after periods of intense activity (e.g., teaching all day) may not manifest during SLP pre- and post-evaluation sessions (Grillo, 2011). Therefore, there is a need to adapt a different model of vocal monitoring that is more frequent. Such a model will also need to be accessible and user-friendly. Some recent studies have demonstrated ambulatory monitoring of voice, albeit in a research setting (Hunter, 2012; Mehta et al., 2013, 2015). These studies typically involve the use of specialized equipment that are expensive and are only available to researchers.

One solution that is readily available and easy to use involves applications (apps) downloaded to smartphones. Smartphones and apps are a part of everyday life and will continue to increase in presence over the next decade. Projections for 2013–2017 suggest that smartphone use will rise from 61.1% to 69.4% globally with 1.75 billion people using such devices by 2014 (eMarketer, 2014). As of September 2014, 71% of people in the USA own a smartphone with 85% of millennials (i.e., people aged 18–24) owning the devices (Nielson, 2014). The weekly time spent using apps has increased from 23 hours in 2012 to 37 hours in 2014, a 63% rise in just two years (Nielsen, 2015). Apps that run on mobile devices offer software solutions that extend the reach and productivity of a typical data collection session that is completed in-person with an SLP.

There are numerous voice or speech recording apps that run on iOS, Android, and Windows platforms. For the purposes of voice and speech analysis, apps that record.wav files at a sampling rate of 44,100Hz are sufficient (Plichta & Kornbluh, 2002). The SLP could require that the patient record his/her voice before and after talking for the day using the app on the smartphone and email the files to the SLP. The SLP could then analyze those files on software that is typically used for voice analysis (e.g., Praat). The ease of access of recording the voice throughout a day of talking via the smartphone will provide realistic data that better represents the effects of vocal loading on the voice.

Previous work has suggested that sound measurement apps for Apple smartphones may be considered accurate and reliable for assessing occupational noise exposure (Kardous & Shaw, 2014) and correlations of acoustic measures taken simultaneously from a head mounted microphone and a Samsung Galaxy Note 3 were significant and strong (r = 0.73, Uloza et al., 2015). The purpose of the current pilot study was to compare within-subject variability among voice measures with different recording devices (i.e., head mounted microphone, Apple, and Android smartphones) and software (i.e., ADSV, MDVP, and Praat). In addition, correlations among voice software programs that provided the voice analysis were also assessed.

## METHODS

**Figure 1:**
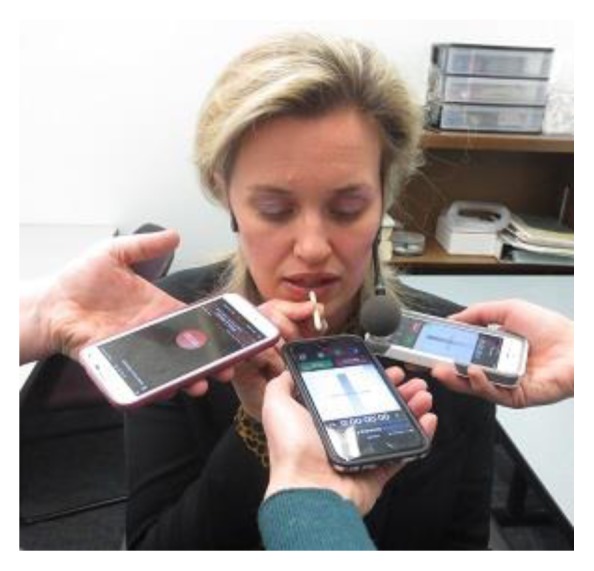
The experimental set-up with the recording devices (iPhone 5 and 6s, Samsung Galaxy S5, head mounted microphone) and the plastic stick that measured 4 cm from the mouth to the microphones.

Ten vocally healthy women and men produced three trials of /a/ sustained for five seconds and three trials of “we were away a year ago” at a comfortable fundamental frequency (F_0_) and intensity. “We were away a year ago” was selected because all the phonemes are voiced, providing a connected speech example of continuous vocal fold vibration. The vocal health of the participants was determined perceptually during conversational speech on the day of testing by the researchers. Each trial was separated by 10 seconds. A head mounted condenser microphone (AKG C420, Northridge, CA), iPhones 5 and 6s, and Samsung Galaxy S5 were placed 4 centimeters (cm) from the participant’s mouth for voice recording (see Figure 1). A 4 cm plastic stick was used to measure the distance from mouth to microphones. All utterances were recorded simultaneously on all devices. Three apps, RØDE Rec LE (iPhone 5) and Recordium (iPhone 6s) for Apple, and Smart Voice Recorder (Samsung Galaxy S5), recorded.wav files. These apps were free, allowed email of the recorded.wav files, and offered a 44,100 Hz sampling rate for recording. The.wav files from the head mounted microphone were saved directly onto the computer that performed the analysis. The middle portion of /a/ (i.e., four seconds, 0.5 seconds trimmed off the beginning and end) and the entire sentence were analyzed.

The acoustic analysis was completed using Praat (Boersma & Weenink, 2015), free software on the web, and KayPENTAX’s (Montvale, NJ) Multi-dimensional Voice Program (MDVP) and Analysis of Dysphonia in Speech and Voice (ADSV). The measures of interest included: fundamental frequency (F_0_), standard deviation of the F_0_ (SD of F_0_), jitter%, shimmer%, noise-to-harmonics ratio (NHR), cepstral peak prominence (CPP), and Acoustic Voice Quality Index (AVQI, Maryn, De Bodt, & Roy, 2010) (see Table 1). The acoustic measures of F_0_, SD of F_0_, jitter%, shimmer%, and NHR were chosen because they represent time-based measures of voice in frequency and amplitude from a nearly periodic voice signal and are measured accurately through sustained vowel. CPP was chosen because it is an alternative to time-based measures and it can be applied to continuous speech, which may provide a more representative sample of voice as compared to sustained vowel. In addition, all of these measures, except AVQI, are among some of the minimum instrumented measures recommended by the Special Interest Group (SIG) 3 Voice and Voice Disorders of the American Speech Language Hearing Association (ASHA) for completion of a comprehensive voice evaluation.

## RESULTS

The main effects of software, device, utterance, and trial were analyzed along with two- and three-way interactions for both women and men participants. For F_0_ and SD of F_0_, the main effect of utterance was significant for women (F_0_ *p* <0.001 and SD of F_0_ *p* <0.001), indicating that F_0_ and SD of F_0_ were different for /a/ and the sentence. No significant other main effects or interactions were found. For men, all main effects and interactions for F_0_ were not significant. The differences in F_0_ seen for women across sustained /a/ and the sentence were not carried over in men. Perhaps with the lower F_0_s seen in men, distinctions between sustained phonation and connected speech were not apparent in this study. That is, with added mass to the vocal folds in men there may be no significant difference in F_0_ for the different speech tasks (i.e., vowel vs. connected speech). For SD of F_0_ in men, the main effects of software and utterance were significant (*p* <0.001). There was also a significant two-way interaction between software and utterance (*p* <0.001). No other significant main effects or interactions were seen for SD of F_0_ in men. The variability around the mean for F_0_ in men did demonstrate differences across sustained phonation and connected speech.

For jitter% and shimmer% in women, main effects for software (*p* < 0.001), devices (*p* < 0.001), and the two-way interaction between software and devices (*p* < 0.001 for jitter% and *p* = 0.01 for shimmer%) were significant. For jitter% in men, main effects for software (*p* < 0.001) and trial (*p* = 0.01) were significant; however, no interactions were significant. For shimmer% in men, the main effect for devices (*p* = 0.01) was significant. No other main effects or interactions were seen.

For NHR in women and men, main effects for software (*p* < 0.001 for women and *p* = 0.05 for men) and devices (*p* < 0.001) were significant, but all two- and three-way interactions were not significant.

For CPP in women and men, the main effects for software, devices, and utterance were all significant (*p* < 0.001) and the two-way interaction for software and devices was significant (*p* < 0.001 for women and *p* = .04 for men). In addition for men, the main effect for trial was significant (*p* < 0.001). Across women and men for CPP, no other main effects or interactions were significant.

For AVQI, software was not a main effect because Praat is the only program that analyzes AVQI. The main effect for devices was significant in both women and men (*p* < 0.001 for women and *p* = 0.01 for men). The other main effect of trial and the two-way interaction of devices and trial were not significant for both women and men. Means and standard deviations for all dependent variables are presented in Tables 1 and 2.

Correlations between software yielded the following results. There was a strong correlation between CPP values calculated by Praat and ADSV for women (r = 0.96, *p* < 0.00) and for men (r = 0.94, *p* < 0.001). For women, there were additional strong correlations between jitter% and NHR calculated by Praat and MDVP (r = 0.64, *p* < 0.001 for both). Shimmer% in women was not a strong correlation between Praat and MDVP (r = 0.11, *p* = 0.07). For men, there were no additional strong correlations (jitter% r = .198, *p* < 0.001; NHR r = 0.29, *p* < 0.001; shimmer% r = 0.12, *p* = 0.04).

## DISCUSSION

Within-subject for both women and men, iPhone 5 and 6s, Samsung Galaxy S5, and the head mounted microphone yielded no significant differences when comparing voice analysis for F_0_, SD of F_0_, jitter%, shimmer%, NHR, CPP, and AVQI across MDVP, ADSV, and Praat. This result is supported by no significant three-way interactions of software, device, and trial indicating that there was no change in the dependent variables across software and across device from trial one to trial three. In addition, algorithms differ for calculating jitter%, shimmer%, NHR, and CPP across software. Even with the different algorithms, there was a strong correlation between ADSV and Praat for calculating CPP in both women and men and also between MDVP and Praat for calculating jitter% and NHR in women only. The overall values may be different, but the trends for these measures follow similar trajectories. It is interesting to note that jitter% and NHR were not strongly correlated across MDVP and Praat for men. Perhaps the lower F_0_s are disrupting the relationship between the algorithms. There was no difference between women and men for CPP because it is not a time-based measure.

The current results are consistent with previous work that suggested certain apps may be used to accurately and reliably measure environmental noise (Kardous & Shaw, 2014) and a Samsung Galaxy Note 3 compared with a head mounted microphone produced strong correlations between acoustic voice measures (Uloza et al., 2015). A recent study presents contradictory suggestions that the use of apps for dB readings of the human voice is premature because all of the three apps tested were not comparable to a Larson-Davis (Depew, NY) Model 831 Type 1 sound level meter (SLM) (Fava, Oliveira, Baglione, Pimpinella, & Spitzer, 2016). Results indicated that three SLM apps on an iPhone 5 and a RadioShack (Fort Worth, TX) SLM yielded inconsistent dB readings for the human voice at soft, habitual, and loud when compared with a Type 1 SLM. Frankly, it is not surprising that the results in Fava and colleagues (2016) were significantly different across recording devices for the human voice recordings and outside of the established criterion of ± 2dB. The procedures did not account for within subject variability across trials. For example, participants only produced one trial of soft /a/ sustained for five seconds. Because the microphones are different across devices, it is expected that the mean results will vary. In fact, the results from the current study were similar to Fava and colleagues (2016) when only looking at the main effect of device. In the current study, there were differences in the means of some of the voice measures across the smartphones and the head mounted microphone. The clinically relevant question is related to maintaining microphone recording integrity across trials in the same individual. The current study addressed that question and found that the smartphones and the head mounted microphone tested enabled consistent analysis of the voice measures within subject across women and men.

Considering the results of this pilot study, it is possible to capture reliable daily vocal loading effects using smartphones and free apps. To limit variability, use the same phone and the same app within each individual and require a 4 cm distance from mouth to microphone. The results are applicable to the phones and the apps used in the study. Future work needs to investigate other phones and other apps, especially given the rapid evolutions in smartphones. If the SLP does not have access to KayPENTAX’s software (i.e., MDVP and ADSV), the recommended minimum acoustic instrumented measures by SIG 3 of ASHA can still be completed using Praat, a free software program downloaded from the internet. In addition, the SLP can include AVQI, which is a measure that is only calculated through Praat. CPP measured through Praat is highly correlated to CPP measured though ADSV for both women and men. Jitter% and NHR are also highly correlated between MDVP and Praat for women only. Even with the measures that are not highly correlated between Praat and ADSV or Praat and MDVP, what matters is within-person change. Differences seen in that individual from pre- to post-treatment carries the most weight regardless of the software program used to perform the analysis. The SLP can complete an acoustic voice evaluation, representing the daily effects of vocal loading, using accessible and low-cost options.

## Figures and Tables

**Table 1:** Acoustic Measures, Definition, Task, and Software

Acoustic Measures	Definition	Task	Software
F_0_	Lowest frequency of periodic vocal fold vibration.	/a/ “we were away a year ago”	Praat and MDVP
SD of F_0_	Variability of the F_0_.	/a/ “we were away a year ago”	Praat and MDVP
Jitter%	Average absolute difference between consecutive periods divided by the average period.	/a/	Praat and MDVP
Shimmer%	Average absolute difference between the amplitudes of consecutive periods divided by the average amplitude.	/a/	Praat and MDVP
NHR	The amplitude of noise relative to tonal components.	/a/	Praat and MDVP
CPP	A measure of the amplitude of the cepstral peak corresponding to the fundamental period, normalized for overall signal amplitude.	/a/ “we were away a year ago”	Praat and ADSV
AVQI	Weighted combination of six time-, frequency-, and quefrequency-domain metrics.	/a/ combined with “we were away a year ago”	Praat

**Table 2:** Means and Standard Deviations (in Parentheses) of the Dependent Variables for Women across Utterance (i.e., /a/ and “we were away a year ago”), Software (i.e., Multi-dimensional Voice Program (MDVP), Analysis of Dysphonia in Speech and Voice (ADSV), and Praat), and Recording Device (i.e., iPhone5 (iPh5), iPhone6s (iPh6s), Samsung Galaxy5 (SG5), and Head-mounted Microphone (HeadMic)

Software	Acoustic Measure	iPh5	iPh6s	SG5	Head Mic
MDVP	F_0_ (Hz) /a/	223.26	223.40	223.44	223.54
	F_0_ (Hz) sentence	200.87	201.02	199.29	201.06
	SD of F_0_ /a/	3.42	2.59	4.31	3.42
	SD of F_0_ sentence	36.03	32.86	36.13	33.61
	Jitter: local(%) /a/	1.11 (0.37)	0.95 (0.33)	2.15 (0.59)	0.96 (0.61)
	Shimmer: local(%) /a/	4.08 (1.28)	4.52 (1.42)	5.06 (1.56)	2.81 (0.81)
	NHR /a/	0.13 (0.01)	0.14 (0.02)	0.15 (0.03)	0.11 (0.03)
PRAAT	F_0 (_Hz) /a/	218.32	217.67	216.41	219.56
	F_0_ (Hz) sentence	197.24	198.58	195.98	198.87
	SD of F_0_ /a/	6.96	7.29	6.29	6.35
	SD of F_0_ sentence	33.91	32.93	33.17	32.96
	Jitter: local(%) /a/	0.41 (0.16)	0.39 (0.13)	0.58 (0.17)	0.41 (0.15)
	Shimmer: local (%) /a/	3.06 (1.26)	5.29 (1.73)	4.38 (1.25)	2.29 (0.52)
	NHR /a/	0.01 (0.00)	0.05 (0.17)	0.03 (0.02)	0.01 (0.00)
	CPP (dB) /a/	24.4 (1.93)	24.1 (1.84)	23.2 (1.81)	24.4 (1.94)
	CPP (dB) sentence	22.9 (2.08)	22.3 (2.15)	20.2 (1.76)	22.9 (2.24)
	AVQI	2.36 (0.49)	3.07 (0.53)	3.68 (0.76)	1.97 (0.31)
ADSV	CPP (dB) /a/	11.1 (1.11)	10.1 (1.01)	9.66 (0.90)	10.1 (1.01)
	CPP (dB) sentence	8.82 (1.03)	7.84 (0.85)	7.17 (0.80)	7.91 (0.83)

**Table 3:** Means and Standard Deviations (in Parentheses) of the Dependent Variables for Men across Utterance (i.e., /a/ and “we were away a year ago”), Software (i.e., Multi-dimensional Voice Program (MDVP), Analysis of Dysphonia in Speech and Voice (ADSV), and Praat), and Recording Device (i.e., iPhone5 (iPh5), iPhone6s (iPh6s), Samsung Galaxy5 (SG5), and Head-mounted Microphone (HeadMic)

Software	Acoustic Measure	iPh5	iPh6s	SG5	Head Mic
MDVP	F_0_ (Hz) /a/	109.44	109.51	109.46	109.44
	F_0_ (Hz) sentence	107.68	108.32	107.59	108.18
	SD of F_0_ /a/	1.74	2.10	1.98	1.76
	SD of F_0_ sentence	10.08	12.92	10.39	12.17
	Jitter: local(%) /a/	1.12 (0.86)	1.12 (0.90)	1.46 (0.84)	1.15 (0.72)
	Shimmer: local(%) /a/	6.71 (4.64)	6.17 (3.49)	5.73 (3.51)	5.08 (4.47)
	NHR /a/	0.17 (0.04)	0.17 (0.05)	0.15 (0.03)	0.13 (0.4)
PRAAT	F_0_ (Hz) /a/	109.41	109.41	109.51	109.50
	F_0_ (Hz) sentence	113.42	110.95	110.69	108.44
	SD of F_0_ /a/	1.33	1.38	1.84	1.84
	SD of F_0_ sentence	28.66	21.52	19.65	13.48
	Jitter: local(%) /a/	0.82 (0.76)	0.87 (0.86)	0.91(0.88)	0.88 (0.97)
	Shimmer: local(%) /a/	5.87 (3.35)	5.85 (2.81)	4.67 (3.05)	3.55 (3.33)
	NHR /a/	0.10 (0.12)	0.09 (0.17)	0.11 (0.24)	0.03 (0.05)
	CPP (dB) /a/	28.97 (2.56)	28.51 (2.36)	27.76 (2.54)	28.67 (2.62)
	CPP (dB) sentence	24.82 (1.78)	24.24 (1.72)	23.06 (1.70)	24.91 (1.80)
	AVQI	2.54 (1.15)	3.00 (1.09)	3.05 (1.17)	2.18 (1.23)
ADSV	CPP (dB) /a/	13.78 (1.98)	12.52 (1.67)	12.41 (1.57)	12.76 (1.77)
	CPP (dB) sentence	9.84 (1.43)	8.71 (1.57)	8.24 (1.65)	8.86 (1.64)
